# Chronic Indwelling Urinary Catheter Increase the Risk of Bladder Cancer, Even in Patients Without Spinal Cord Injury

**DOI:** 10.1097/MD.0000000000001736

**Published:** 2015-10-30

**Authors:** Chung-Han Ho, Kuan-Chin Sung, Sher-Wei Lim, Chien-Hwa Liao, Fu-Wen Liang, Jhi-Joung Wang, Chia-Chun Wu

**Affiliations:** From the Department of Medical Research, Chi Mei Medical Center, Tainan City, Taiwan (C-HH, J-JW); Department of Hospital and Health Care Administration, Chia Nan University of Pharmacy and Science, Tainan City, Taiwan (C-HH); Department of Pharmacy, Chia Nan University of Pharmacy and Science, Tainan City, Taiwan (C-HH, C-CW); Department of Neurosurgery, Chi Mei Medical Center, Tainan City, Taiwan (K-CS); Department of Neurosurgery, Chi Mei Hospital, Chiali, Tainan City, Taiwan (S-WL); Department of Nursing, Min-Hwei College of Health Care Management, Tainan City, Taiwan (S-WL); Institute of Biomedical Sciences, National Sun Yat-sen University, Kaohsiung City, Taiwan (S-WL); Division of Urology, Department of Surgery, Chi Mei Medical Center, Tainan City, Taiwan (C-HL); Department of Senior Citizen Service Management, Chia Nan University of Pharmacy and Science, Tainan City, Taiwan (C-HL); Department of Public Health, College of Medicine, National Cheng Kung University, Tainan City, Taiwan (F-WL); and Department of Nephrology, Chi Mei Medical Center, Tainan City, Taiwan (C-CW).

## Abstract

Chronic indwelling urinary catheters (CIDCs) are known as a risk factor for bladder cancer in patients with spinal cord injury (SCI). This study examined the potential risk of bladder cancer from CIDCs in patients without SCI.

The National Health Insurance Research Database in Taiwan was used to identify SCI patients (N = 1816). This group was compared against a control CIDC cohort without SCI (N = 1816) and a reference cohort with normal individuals without SCI and a record of CIDC (N = 7264). Comparisons were made based on age and gender matching over a maximum of 11 follow-up years. The incidence risk and hazard ratio (HR) of bladder cancer were estimated in all 3 groups.

During the follow-up period, the bladder cancer incidence rates were 68.90 and 102.53 per 100,000 person-years in the SCI and CIDC-non-SCI groups, respectively. These values were both higher than that of the reference cohort (12.00 per 100,000 person-years). Patients who had history of SCI (HR: 6.51; 95% CI, 2.56–16.52) or CIDC without SCI (HR: 9.11; 95% CI, 3.9–21.29) had a higher risk of bladder cancer compared with the reference cohort.

Patients with CIDCs may have an increased risk of bladder cancer development, especially in older aged and male patients compared with general population.

## INTRODUCTION

Bladder cancer is the 7th most common malignancy and the 9th most common cancer site that results in death in males worldwide.^1^ Bladder cancer is often found in males, especially in older men. The known risk factors of bladder cancer are smoking, toxic chemical exposure, and prolonged cyclophosphamide therapy.^2,3^ Spinal cord injury (SCI) is also a reported risk factor for bladder cancer, especially the squamous cell type.^4^ Although patients with SCI are known as a high-risk group for bladder cancer, no controlled prospective study has ever been published due to its low incidence rate. Several studies have indicated that the relative risk of bladder cancer in SCI patients is higher than in the general population, but most of these studies were descriptive or cross-sectional studies.^5–7^ Additionally, a few cohort studies have presented conflicting results.^6,8–10^ Groah et al^8^ showed that their SCI population had a 25.4-fold increase risk of bladder cancer compared with an age- and gender-adjusted normal population. However, some studies indicated that the bladder cancer incidence was not significantly different between SCI patients and the general population.^9,10^ Chronic indwelling catheter (CIDC) use is an important risk factor of bladder cancer development in SCI patients.^6,8,11–13^ However, no study has reported the bladder cancer incidence from CIDC in patients without SCIs. In this study, we wanted to evaluate if CIDC itself is an independent risk factor of bladder cancer by comparing the bladder cancer incidence in patients with CIDCs but without SCI (CIDC non-SCI) and the general population without CIDCs. Furthermore, we also compared the bladder cancer incidence between CIDC patients without SCI and SCI patients to determine if SCI patients have an even higher bladder cancer risk under an immune depression status.

## MATERIALS AND METHODS

The National Health Insurance Administration of the Ministry of Health and Welfare in Taiwan provided the National Health Insurance Research Database (NHIRD). The dataset included all claims data from Taiwan's National Health Insurance program since 1997. Additionally, approximately 99% of the population in Taiwan has enrolled in the National Health Insurance program. All of the patients receiving a diagnosis in Taiwan were recorded in this database. The diseases recorded in this database were consistent with the International Classification of Diseases format, Ninth Revision, Clinical Modification (ICD-9CM) code. The drug prescriptions and the operation recodes were based on the medical expenditure applications. Informed consent was originally obtained by the National Health Research Institutes in Taiwan. Because the privacy of each individual's information was protected using encrypted personal identification to avoid the potential for ethical violations that were related to the data, informed consent was not required. Exemption was obtained from the institutional review board of Chi Mei Medical Center (IRB No. 10203-E02).

### Study Subjects

In this study, data for patients with SCI, paraplegia, or quadriplegia were extracted with the ICD-9CM codes 806 and 952 for catastrophic illness certification. The purpose of combining catastrophic illness certification is to exclude the less severe SCI patients with preserved bladder function, if possible.

The comparison control cohort was applied from the longitudinal health insurance database in year 2000 (LHID2000), which is an NHIRD subset database. Patients who had CIDCs but did not have SCI were 1:1 selected as the CIDC-non-SCI group. The CIDC patients included those who used a CIDC more than 6 times. Patients with any malignancy other than bladder cancer and diabetes mellitus were excluded from the study. Patients with multiple sclerosis (ICD-9CM:340), autoimmune disease: systemic lupus erythematosus (ICD-9CM:710.0); rheumatoid arthritis (ICD-9CM:714); vasculitis (ICD-9CM:446.4), or glomerulopathy (ICD-9CM:580–589 combined with renal biopsy ICD-9CM:55.23), as well as organ transplant recipients (ICD-9CM:E878.0), and those who were possibly treated with cyclophosphamide and any history of cyclophosphamide exposure from the medical expenditure applications were also excluded. Because one of our study aims was to determine the bladder cancer incidence ratio between patients with or without SCI, the potential risk factors that lead to bladder cancer were excluded. The other exclusion criteria pre-SCI or pre-index date included bladder stones (ICD-9CM:594.1), cerebrovascular accidents (ICD-9CM:430–438), congenital anomalies (ICD-9CM:753.6 and 753.8), and bladder obstruction history (ICD-9CM:596.0). For the general control population, we also excluded patients who had ever had a CIDC based on medical expenditure applications. For the observational data to be considered for the bladder cancer incidence, the cases were selected from 1998 through 2002. For each SCI and CIDC-non-SCI patient, four patients were selected as controls. The controls were matched by age and gender and were without an SCI diagnosis and CIDC record during the whole study period.

### Outcome Variables

The outcome of interest was bladder cancer occurrence (ICD-9CM:188). The patients were right censored on December 31, 2008, or on the date they were issued a catastrophic illness card for bladder cancer, died, withdrew from the insurance program, or were lost to follow-up. The patients were followed up for a maximum of 11 years.

### Statistical Analysis

Mean differences in continuous variables were analyzed by analysis of variance, and Pearson Chi-square test was applied for categorical variables. The incidence rate of bladder cancer in each group was computed as the counts of bladder cancer patients divided by the total number of following years during the study period. Absolute risk estimates were calculated as rates per 100,000 person-years. The incidence rate ratio of bladder cancer was estimated using the Poisson regression with offset variable, total person-time. Additionally, the probability of bladder cancer free for each group was plotted by the Kaplan–Meier method, and the difference was examined by the log-rank test. The selected risk factors were also estimated with Cox regression analyses. Analyses were adjusted by including the described variables as covariates in the Cox models. A *P*-value of <0.05 was considered statistically significant. The statistical software Statistical Analysis System (SAS) (version 9.4; SAS Institute, Inc., Cary, NC) was used to perform all of the statistical analyses.

## RESULTS

A total of 1816 patients in our study population had a history of SCI with severe sequela and had obtained a catastrophic illness certification between 1998 and 2002. Another 1816 1:1 age–sex-matched CIDC-non-SCI group patients and 7264 control group patients were selected in our study. The mean age of our study population was 47.42 ± 15.84 years old, and approximately 70% of these patients were male. In the CIDC-non-SCI group, acute urine retention and neurogenic bladder diagnosis codes contributed to 30.8% of the study population, cerebrovascular accidents and dementia contributed to 20.7% and benign prostatic hyperplasia (BPH) contributed to 9.1% of all the diagnosis codes. On average, the SCI patients had higher death rates than the CIDC-non-SCI group and control group patients (Table 1).

**TABLE 1:**
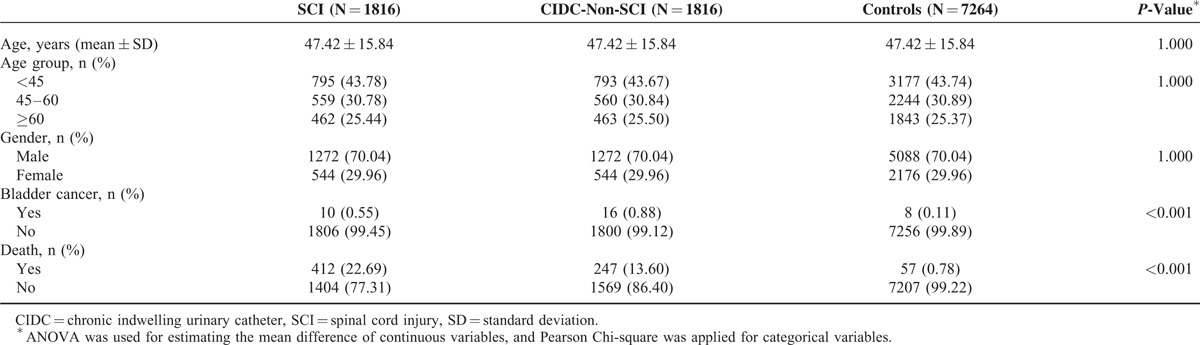
Demographic Characteristics of Spinal Cord Injury Patients, Chronic Indwelling Urinary Catheters Patients Without Spinal Cord Injury, and General Population Controls

During the follow-up period, 0.55% (10/1816) and 0.88% (16/1816) of the SCI and with CIDC-non-SCI had new diagnosis of bladder cancer, in contrast to 0.11% (8/7264) in control group. The bladder cancer incidence rates were 68.90 and 102.53 per 100,000 person-years in the SCI and CIDC-non-SCI groups, respectively, which were both higher than that in the control group (12.00 per 100,000 person-years). Overall, the patients who were older or male had a higher risk of bladder cancer (Table 2).

**TABLE 2:**
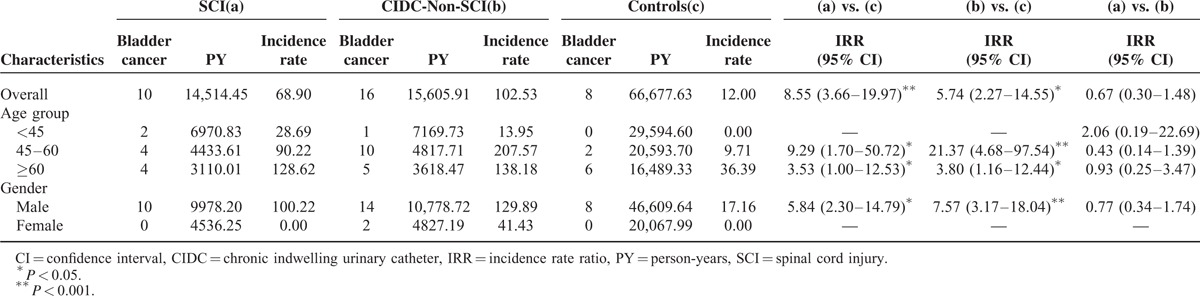
Incidence of Bladder Cancer in Spinal Cord Injury Patients, Chronic Indwelling Urinary Catheters Patients Without Spinal Cord Injury, and General Population Controls

The SCI and CIDC-non-SCI group patients had a bladder cancer incidence that was 5.74 and 8.55 times greater than the incidence in the control group patients, respectively (Table 2). Of the patients between 45 and 60 years old, the SCI and CIDC-non-SCI groups had a 9.29-and 21.37-fold higher risk of bladder cancer than those in the control group, respectively. Of the patients who were ≥60 years old, the bladder cancer risk was also significantly higher in the SCI and CIDC-non-SCI groups than that in the control group (ratios of 3.53 and 3.8, respectively). Of the male patients, the bladder cancer risk was 5.84- and 7.57-fold higher in the SCI and CIDC-non-SCI groups than in the control group (Table 2). However, the overall bladder cancer risk was not significantly different between the SCI and CIDC-non-SCI group patients.

**FIGURE 1:**
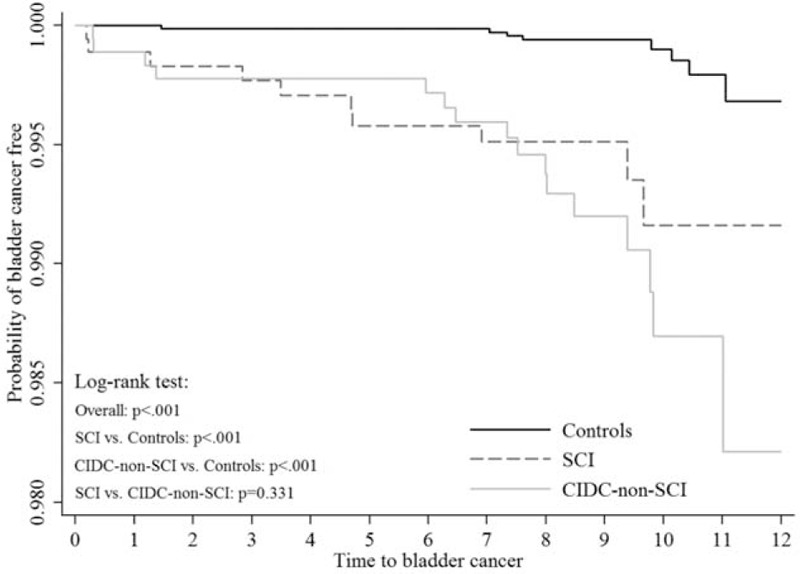
Time-to-event curves by study groups for new diagnosis of bladder cancer. Event rate represent Kaplan–Meier estimates; log-rank test: *P* < 0.0001 for overall difference, *P* < 0.0001 for SCI vs. controls, *P* < 0.0001 for CIDC-non-SCI vs. controls, and *P* = 0.3313 for SCI vs. CIDC-non-SCI.

In our total study population, a history of SCI (hazard ratio (HR), 6.51; 95% CI, 2.56–16.52) and CIDC without SCI (HR, 9.11; 95% CI, 3.9–21.29) were associated with higher risk of bladder cancer when compared with the general population (Table 3). The Kaplan–Meier plots showed that the SCI and CIDC-non-SCI group patients had a higher risk of bladder cancer development than the general population; however, no significant differences were identified between the SCI and CIDC-non-SCI groups (Fig. 1). Amongst the 16 patients who developed bladder cancer in the CIDC-non-SCI group, 1 had a urinary tract infection history, 2 had urolithiasis instances, and none had BPH (Table 4).

**TABLE 3:**
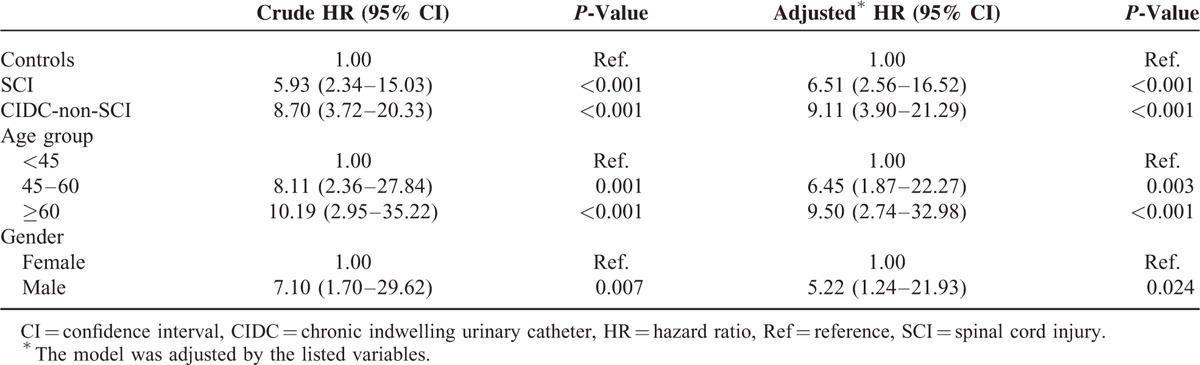
Hazard Ratio of Bladder Cancer Development in Spinal Cord Injury Patients and Chronic Indwelling Urinary Catheters Patients Without Spinal Cord Injury Compared With General Population Controls

**TABLE 4:**
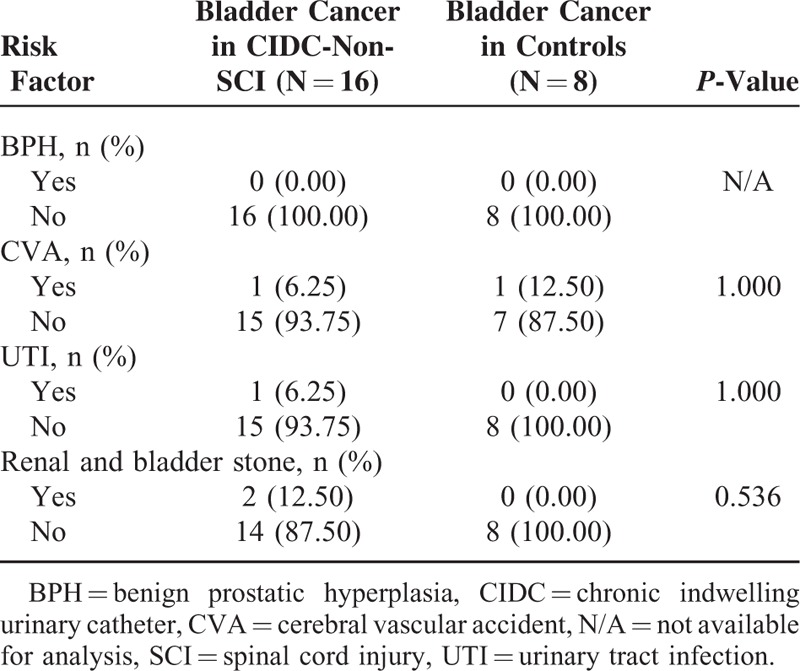
The Bladder Cancer Risk Factor Distribution Between the CIDC-Non-SCI and General Population Controls

## DISCUSSION

Our study results showed that the CIDC-non-SCI group patients had an increased risk of bladder cancer compared with the general population, and the risk was even higher if they were younger than 60 years of age. SCI patients also had a higher risk of bladder cancer compared with the general population; however, the risk was not significantly different compared with the CIDC-non-SCI patients.

To the best of our knowledge, this is the first study to determine that CIDC could also be a risk factor for bladder cancer in patients without SCI. The inflammatory microenvironment is known as a critical cause of tumor formation.^14^ According to epidemiology studies, infection, inflammation caused by irritants (eg, urinary tract stones and indwelling catheters in SCI patients), cyclophosphamide-induced cystitis, BPH, and non-steroidal anti-inflammatory drugs were all risk factors for bladder cancer.^15^ Urinary tract infection is the most common CIDC complication, and bacteriuria is an almost universal finding in CIDC patients (either symptomatic or asymptomatic).^16–18^ Yamamoto et al^19^ and Kawai et al^20,21^ demonstrated that urinary bladder carcinogenesis could be enhanced by heat-killed *E. coli* or lipopolysaccharide-induced chronic inflammation. These findings may partially explain the higher risk of bladder cancer in CIDC patients. Additionally, BPH was found as a risk factor of bladder cancer in a few studies,^22,23^ and incomplete urinary bladder emptying was proposed as a possible mechanism.^24^ Lack of physical activity was also reported to be associated with bladder cancer.^25^ Incomplete urinary bladder emptying in CIDC conditions and low physical activity in cerebral vascular accident or dementia patients might also contribute to the findings of our study results.

Our study indicated that the risk of bladder cancer in SCI patients was higher than the general population. In SCI patients, gross hematuria occurs rapidly after SCI, and this phenomenon is also observed in experimental rats. Histologic changes and tight junction protein expression alterations in the urinary bladder were demonstrated both in acute and chronic stages in rats with SCI.^26,27^ Histologic changes in the urinary bladder after SCI may cause patients to become susceptible to chronic bladder infections and inflammation, which can be followed by bladder cancer. Several studies indicated that CIDC is an important risk factor of bladder cancer in SCI patients.^5,6,8^ However, Kalisvaart et al^28^ reported that neurogenic bladder rather than CIDCs is a bladder cancer risk factor in SCI patients. Despite the immune-suppressive status in SCI patients, our data showed no significant differences in the bladder cancer risk between the observed SCI and CIDC-non-SCI patients. Similar chronic bladder inflammation conditions, including incomplete bladder emptying and low physical activity in SCI patients, may explain the increased bladder cancer risk.

There are several limitations to this study, which would be expected issues when using the administrative database. First, some bladder cancer risk factors, such as smoking and occupations that might result in exposure to aromatic amines, were unavailable. Second, the effect of the indwelling catheter duration on bladder cancer could not be evaluated because of the lack of the exact dates of the first indwelling catheter among the CIDC-non-SCI patients. Third, the bladder cancer and urolithiasis numbers might be underestimated if they were asymptomatic and not diagnosed. Despite these limitations, our study has several strengths. First, this is the first cohort study to evaluate the bladder cancer risk in CIDC patients without a history of SCI. In addition, our study is based on a nationwide administrative database, which includes a large sample size and detailed medical information; therefore, we can exclude other potential bladder cancer risk factors by using the well-matched control and SCI groups to minimize the selection bias.

In conclusion, our study demonstrated that CIDCs is associated with the increased risk of bladder cancer even in patients without SCI which might result from the chronic inflammation caused by CIDCs and that the substantially elevated risk is not inferior to that in SCI patients; however, the real mechanisms need to be clarified. We advocate avoiding a CIDC as much as possible. Future improvements in urinary catheter designs and care are expected to decrease chronic inflammation in patients for whom CIDCs is inevitable.
